# Adenosquamous Carcinoma with the Acantholytic Feature in the Oral Cavity: A Case Report and Comprehensive Literature Review

**DOI:** 10.3390/diagnostics12102398

**Published:** 2022-10-02

**Authors:** Tatsuya Abé, Manabu Yamazaki, Satoshi Maruyama, Nobuyuki Ikeda, Yoshimasa Sumita, Kei Tomihara, Jun-ichi Tanuma

**Affiliations:** 1 Division of Oral Pathology, Faculty of Dentistry & Graduate School of Medical and Dental Sciences, Niigata University, Niigata 951-8514, Japan; 2 Oral Pathology Section, Department of Surgical Pathology, Niigata University Hospital, Niigata 951-8520, Japan; 3 Oral and Maxillofacial Surgery, Faculty of Dentistry & Graduate School of Medical and Dental Sciences, Niigata University, Niigata 951-8514, Japan

**Keywords:** adenosquamous carcinoma, acantholytic squamous cell carcinoma, oral cavity

## Abstract

Adenosquamous carcinoma (ASC) is an aggressive subtype of squamous cell carcinoma (SCC). Due to its poor prognosis, a precise pathological diagnosis of ASC is essential but challenging because its pathological criteria are still unclear. Here, we present a rare case of oral ASC accompanied by acantholytic features. The tumor was raised in the mandibular gingiva and recurred locally approximately 13 months after the initial surgery with cervical lymph node metastasis. Pathological specimens of the primary lesion showed acantholysis in a large area of the SCC. Mucous cells, the characteristic finding indicating glandular differentiation, were imperceptible in the initial surgical specimen but increased in the locally recurrent and metastatic lymph node specimens. In a comprehensive literature review of oral ASC cases, the present case was the only case of ASC with acantholytic features. We reconfirmed that ASC has poor prognoses, such as low 5-year overall survival and disease-free survival, high locoregional recurrence, and high distant metastasis rates. A precise diagnosis of ASC is required for estimating prognosis and undergoing close follow-up, even if the adenocarcinomatous component is limited to a small area in the lesion.

## 1. Introduction

Adenosquamous carcinoma (ASC) in the oral cavity is a rare subtype of squamous cell carcinoma (SCC) [1]. ASC in the head and neck region was first recognized by Gerughty et al. in 1968 [2]. Their report defined ASC as a malignant neoplasm containing unequivocal adenocarcinoma and separate areas that were distinctly SCC [2]. The differentiation of ASC from conventional SCC is necessary because ASC has a poorer prognosis than conventional SCC. ASC can be found in various organs; however, its histopathogenesis in the head and neck region is not fully understood, and its histological diagnostic criteria have not been clearly stated [1].

On the other hand, acantholytic SCC (AcSCC) is also well-recognized as a subtype of SCC [1]. Histologically, AcSCC is characterized by loss of cohesion among neoplastic prickle cells [3]. Lever first described the AcSCC in 1947 [4]. After the first report of cutaneous cases of AcSCC, cases in non-sun-exposed areas, such as the aerodigestive tract, including the oral mucosa [5], have been reported. Morphologically, AcSCC can frequently mimic glandular structures by acantholysis; therefore, it is called adenoid SCC. Despite the closeness of the morphologies between ASC and AcSCC, ASC has a poorer prognosis than AcSCC [6]. Thus, the pathological distinction of ASC from AcSCC is important for estimating patient prognosis.

Here, we report a rare case of ASC with the acantholytic feature and local and regional recurrences and its immunohistochemical profile. Furthermore, a comprehensive review of ASC cases in the oral cavity was performed to clarify their biological behavior.

## 2. Case Presentation

### 2.1. Clinical History

**Figure 1:**
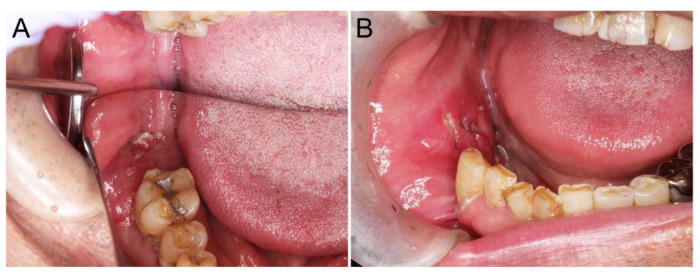
Intraoral findings of adenosquamous carcinoma (ASC) with the acantholytic feature. At initial onset (**A**) and recurrence (**B**). After extracting the second molar of the right side of the mandible, an ulcerative lesion remained, and a large mass expanded to the buccal side of the first molar (**A**). At a recurrence, a submucosal mass with an ulcer was located in the right buccal mucosa (**B**).

A 77-year-old male patient complained of delayed healing after the extraction of the right second molar of the mandible. On his first visit to our hospital, an intraoral examination revealed a deep ulcer with induration, measuring 25 × 20 mm, in the right molar region of the mandible (Figure 1A). No palpable nodules were observed on either side of the neck. Computed tomography (CT) revealed a bone-destructive lesion that expanded from the right molar gingiva to the mandibular bone, measuring 30 × 22 × 31 mm. Magnetic resonance imaging (MRI) revealed that the lesion showed a high signal intensity on T2-weighted imaging. An incisional biopsy specimen was diagnosed as a squamous cell carcinoma of the gingiva. Clinical staging was evaluated as cT4aN0M0, according to the TNM classification, 8th edition of the Union for International Cancer Control (UICC) [7]. Resection of the right side of the mandible to the buccal mucosa and elective neck dissection (level I of the right neck) were performed. A histological examination of the resected specimen revealed a complete tumor resection. No lymph node metastasis was observed, and no postoperative chemoradiotherapy was administered.

A year after the first operation, an ulcerative mass with induration in the right buccal mucosa of the first operation area was identified (Figure 1B). As CT and MRI also suggested local recurrence of cancer, an incisional biopsy was performed, and a diagnosis of squamous cell carcinoma compatible with local recurrence was made approximately 13 months later from the first resection. Positron emission tomography-CT revealed a cervical lymph node metastasis. Surgical resection of the right buccal mucosa and radical neck dissection, and subsequent radiotherapy, were performed. A year and a half after resectioning the recurrent lesion and neck dissection, no local or regional recurrence or distant metastasis were detected.

### 2.2. Pathological Findings

**Figure 2:**
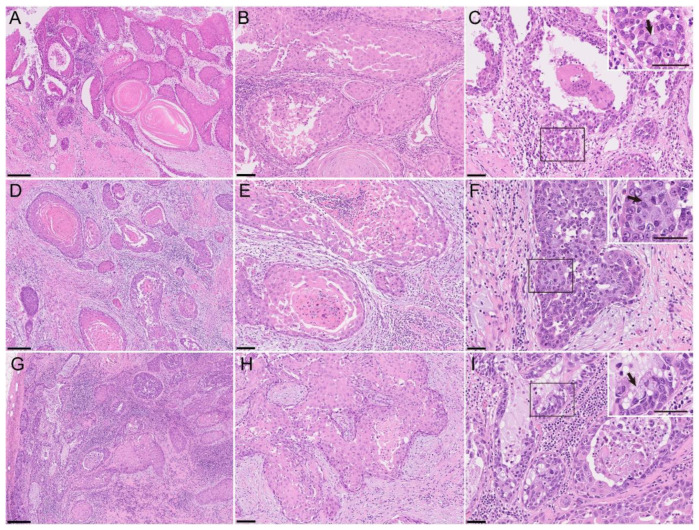
Histology of ASC with the acantholytic feature in the primary (**A**–**C**), recurrent (**D**–**F**), and metastatic (**G**–**I**) lesions. Hematoxylin–eosin (HE) staining (**A**–**I**). Arrows, mucin-containing cells. Bars, 250 μm (**A**,**D**,**G**), 100 μm (**B**,**E**,**H**), and 50 μm (**C**,**F**,**I**). In the primary mandibulectomy specimen, a small glandular/mucinous component was formed in the cancer tissue within the well-differentiated squamous cell carcinoma (SCC) component (**A**). The SCC component shows acantholysis in the glandular/mucinous component (**C**), small cuboidal cells lining the luminal structure, and a small number of cuboidal cells with intracytoplasmic basophilic mucin (arrows). Multinucleated foreign body-type giant cells were present in the luminal space. In the resected buccal mucosa at recurrence, acantholysis of the SCC component is observed in a large part of the SCC (**E**). Less than 5% of cancer cells and the mandibulectomy sections showed glandular or mucinous differentiation. In the glandular component, small basophilic cuboidal cells showed a polarized nuclear location and prominent nucleoli intermingled with goblet-like cells with intracytoplasmic mucin (**F**). The components of SCC with acantholysis and glandular/mucinous components were admixed, even in lymph nodes with cancer metastasis (**H**). The glandular/mucinous component was observed in larger areas of the metastatic lesions to the cervical lymph nodes than in the primary site (**E**). The metastasized carcinoma showed an obvious glandular structure with intraluminal mucin lined by cuboidal cells and mucin-containing cells (**I**).

From the macroscopic findings of the resected sample of the right sectional mandibulectomy, a solid and whitish, poorly demarcated lesion with ulceration of the gingiva expanded to the mandibular bone marrow and fatty tissue surrounding the mandible. Histopathological examination revealed that the lesion predominantly consisted of well-differentiated SCC (Figure 2A), which was associated with an intraepithelial lesion in the gingival mucosa that invaded the mandibular bone marrow and peri-mandibular soft tissues. However, the SCC component largely exhibited acantholysis (Figure 2B). Less than 1% of the tumor area showed a gland-like structure with central tumor cell dissociation (Figure 2C). The gland-like structure consists of basaloid tumor cells, which were characterized by scanty and basophilic cytoplasm and condensed nuclei, and intracytoplasmic-mucin-containing goblet-like tumor cells. There were no findings of vascular and perineural invasion in the specimen. All surgical margins were tumor-free. The pathological classification was evaluated as pT4a according to UICC [7].

The resected specimen of the right buccal mucosa at recurrence was macroscopically covered with smooth and flat mucosa. On gross cutting, a poorly demarcated whitish lesion was observed in the muscular layer of the buccal region and expanded to the subcutaneous tissue. Histologically, well-differentiated SCC was evident; however, most parts of SCC showed acantholysis (Figure 2D). The acantholytic area showed central necrosis (Figure 2E). In addition, the acantholytic area of SCC was continuous with the gland-like structure with low-columnar cells and mucin-containing cells, and it was limited to less than 5% of the tumor (Figure 2F) in the specimen of the first operation. In the metastatic lesion in the neck lymph node, the SCC component with acantholysis and the glandular components, including mucinous cells and intraluminal mucin, were accompanied (Figure 2G). The cellular attachment was lost among the eosinophilic prickle cancer cells (Figure 2H). A gland-like structure with mucin-containing cells was also observed in the metastatic region of the right neck lymph nodes (Figure 2I). Approximately 50% of the tumor tissue of the metastatic lesion consisted of a component that showed a gland-like structure with mucin-containing cells.

**Figure 3:**
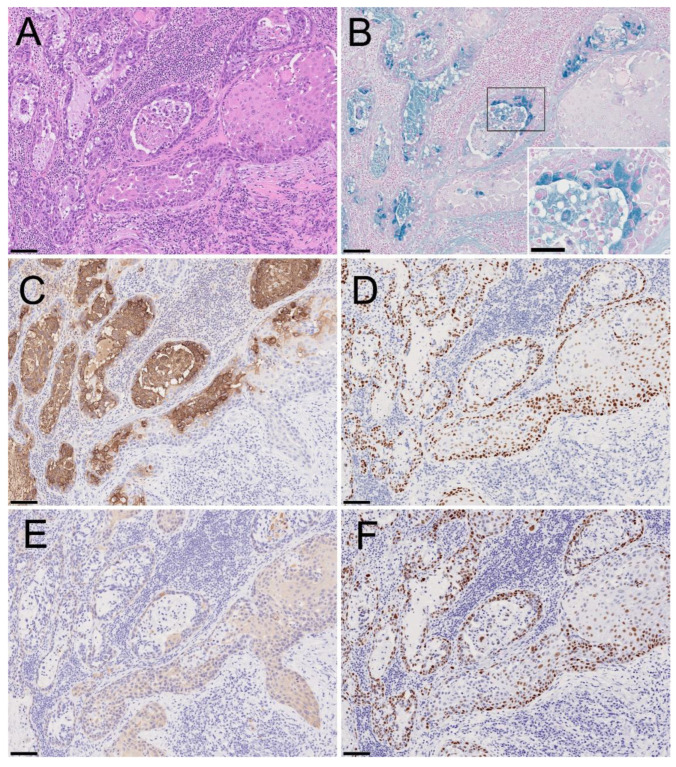
Immunohistochemistry and mucin staining profiles of ASC with the acantholytic feature. HE staining (**A**), Alcian blue (**B**), carcinoembryonic antigen (CEA) (**C**), p40 (**D**), cytokeratin (CK) 5/6 (**E**), and p53 (**F**). Bars, 100 μm (**A**–**F**) and 50 μm (inset). A representative image of ASC in metastasized lymph nodes shows that the glandular component with intraluminal mucin deposition transitioned to the squamoid component with acantholysis (**A**). Alcian blue-positive acidic mucin was observed in the cytoplasm of the goblet-shaped cells and intraluminal mucin (**B**). CEA was strongly positive in the cytoplasm of the cancer cells, consisting predominantly of glandular structures, but also in the dissociated cancer cells in the squamoid component (**C**). Diffuse p40 nuclear positivity was observed in the squamoid and glandular cells, except for mucin-containing cells (**D**). CK5/6 was faintly positive, predominantly in the squamoid component (**E**). Diffuse strong p53 positivity was observed in both components (**F**).

**Figure 4:**
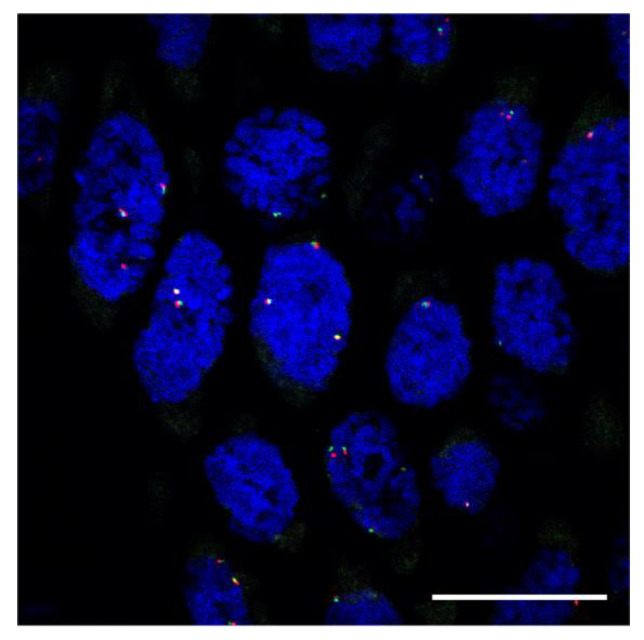
Fluorescent in situ hybridization with *MAML2* break-apart probes of the present case. Bar, 20 μm. In the present case, the tumor cells showed no split signal of *MAML2* probes (overlapped red and green signals). A triple overlap signal in the individual nuclei was frequently observed, which might indicate aneuploidy.

The mucin-containing cells (Figure 3A) showed intracytoplasmic positivity for Alcian blue staining (Figure 3B), indicating acidic mucopolysaccharide production. Immunohistochemically, the mucin-containing cells were positive for carcinoembryonic antigen (CEA) (Figure 3C) and MUC1; they were partly positive for CAM5.2 but not for cytokeratin (CK) 7. In the periphery of the gland-like structure, the tumor cells were also positive for p40 (Figure 3D) and CK5/6 weakly (Figure 3E), but mucin-containing cells were not. These results suggest that mucin-containing cells were associated with glandular differentiation. However, the cancer cells in the glandular components retained the expression of squamous-differentiation-related molecules. The immunopositivity of p53 was strong and diffuse in both parts of the SCC with acantholysis and glandular/mucinous components (Figure 3F). Furthermore, *MAML2* break-apart fluorescence in situ hybridization (FISH) was negative for the present lesion (Figure 4). According to the histological evaluation, the histopathological diagnosis was confirmed as ASC with acantholytic features.

## 3. Discussion

Here, we report a case of oral ASC with acantholytic features. The tumor consisted of a predominant SCC component with acantholytic features and a small amount of a glandular component. ASC with acantholytic features is rare. To our knowledge, no previous reports on the oral cavity exist. ASC associated with acantholysis has been reported in the esophagus [8] and pancreas [9]. The separation of ASC from conventional SCC and AcSCC is considerably important to predict the patient’s prognosis, since ASC shows a poorer prognosis. Both immunohistochemistry for multiple markers for glandular differentiation and special staining to detect mucin are highly recommended for diagnosing ASC.

The histological criteria for ASC of the oral cavity remain unclear. In the WHO classification, the definition of ASC is stated in the section of the tumor of the hypopharynx, larynx, trachea, and para-pharyngeal space as “a malignant tumor that arises from the surface epithelium and shows both squamous and glandular differentiation” [1]. However, the criteria for the amount of glandular differentiation have not been clarified. In our case, the amount of glandular component was considerably small in the primary lesion (<5%); however, it increased in the recurrent and metastatic lesions (up to 50%). This finding suggests that the glandular component is critical to defining the biological behavior of ASC, and the pathological diagnosis of ASC should be considered despite the amount of glandular component.

Although immunohistochemistry can help identify glandular differentiation, it also has limitations. The immunohistochemical positivity of CK7 and CAM5.2 might also support glandular differentiation in various organs [10,11]. However, CK7 was negative, and CAM5.2 was positive only in a small number of cancer cells in the present case. The glandular components of reported cases of oral ASC were typically positive for CK7 [12,13,14,15,16,17,18,19,20,21]. The mucin-containing cells were CEA-positive and p40-negative, similar to the immunohistochemical results for ASC reported by Satomi et al. [15]. These results suggest that mucin-containing cells possess a glandular phenotype, and CK7 is not a definitive marker of ASC in the oral mucosa. In addition, cancer cells located in the periphery of glandular structures were positive for p40, which is a squamous differentiation marker [22]. Immunoreactivity suggested that the squamous phenotype remained on the cancer cells in the glandular structure. For immunohistochemical evaluation, multiple types of intermediate filaments should be examined. In addition, non-neoplastic mucinous cells that remain in the cancer nest due to glandular involvement of cancer cells should be ruled out carefully during the diagnostic process.

SCC with acantholytic features, known as AcSCC [1], should be distinguished from ASC. AcSCC showed histological mimicking of the glandular structure, leading to the mistaking of true glands [23]. Thus, AcSCC is known as adenoid SCC [4]. This histological similarity between ASC and AcSCC/adenoid SCC causes confusion in diagnosis. However, the diagnostic separation of ASC from AcSCC is important because ASC has a poorer prognosis than AcSCC [24]. Adenoid components in AcSCC morphologically mimic glandular differentiation and are negative for mucin [25,26]. Therefore, to differentiate ASC from AcSCC, the identification and detection of mucin are crucial.

Mucoepidermoid carcinoma (MEC) of the salivary glands, including mucosa-associated minor salivary glands, can be a problematic differential diagnosis in the present case. Histopathologically, MEC consists of cancer cells with squamous and mucinous differentiation and “intermediate cells”. In molecular analysis, 56–75% of MEC cases showed rearrangement of the *MAML2* gene [27]. The present case showed biphasic differentiation of cancer cells as well as MEC. However, intraepithelial or dysplastic precursor lesions observed in the present case suggested a mucosal epithelial origin of the tumor. Furthermore, the lack of “intermediate cells” and the negative results of *MAML2* break-apart FISH also supported the diagnosis of ASC.

From our comprehensive literature review of ASC arising in the oral cavity (Table 1), 50 cases, including the present case, have been reported [2,12,13,14,15,16,17,18,19,20,21,23,28,29,30,31,32,33,34,35,36,37], and the clinical information of the review is summarized in Table 2. The median age of the patients was 60 (range 22–97). The sex ratio was approximately 2:1 for male:female. These tendencies are almost equivalent to the conventional SCC [1]. In terms of the site, the tongue is the most frequent primary site. However, the characteristic point was that the second frequent site was the floor of the mouth. This anatomical feature may be related to the distribution of secretory glands associated with the mucosal squamous epithelium.

Our review of the literature reconfirms the aggressiveness of ASC. Locoregional recurrence, local recurrence, regional lymph node metastasis, regional recurrence, distant metastasis rates, and the rate of death of the disease were calculated as 62.8% (27/43 cases), 52.2% (12/23), 46.5% (20/43), 14.0% (6/43), 30.0% (9/30), and 31.1% (13/42), respectively. Similarly, Keelawat et al. [23] reported that 46.7% of patients had experienced local recurrences: 64.7% had positive cervical lymph nodes, 23.1% developed distant metastases, and 42.9% died of their disease at a mean follow-up period of 24.7 months in ASC of the aerodigestive tract, including the oral cavity. Conventional oral SCC also had lymph node metastasis in approximately 40% of patients [38].

However, conventional SCC of the oral cavity showed an overall recurrence rate of 15.5–34.4%, with 5.4–19.9% local and 8.5–14.6% regional [39,40]. In addition, the distant metastasis rate of conventional SCC of the oral cavity is estimated to be 3–9.6% [41,42,43]. In comparing ASC with conventional SCC, unfavorable events, such as locoregional recurrences and distant metastasis, were markedly more frequent in patients with ASC. In terms of the outcome data (Table 1), the 5-year and 3-year overall survival (OS) rates of ASC were 37.1% [95% confidence interval (CI): 15.4–59.0%] and 46.3% [95% CI: 25.1–65.2%], respectively, and the median OS interval was 36 months. The 5-year and 3-year disease-free survival (DFS) rates were 28.0% [95% CI: 10.8–48.3%], and the median DFS interval was 13 months. Schick et al. reported that the 3-year OS and DFS rates were 52% [95% CI: 28–75%] and 32% [95% CI: 11–54%], respectively, while the median OS and DFS intervals were 39 and 12 months, respectively [31]. Meanwhile, the 5-year OS and DFS rates of conventional SCC of the oral cavity were 56–66% and 71–79%, respectively [44,45,46]. Our secondary analysis of the literature data has limitations in the analytical accuracy, since not all the reports we reviewed clarified the starting points of follow-up intervals and the detailed information that would affect the outcome, such as the tumor sizes, tumor stages, or therapeutic modality. The survival of the cancer patient is associated not only with the results of surgical resection but also with the effects and side effects of adjuvant therapy, such as radiotherapy and chemotherapy. Furthermore, the effect of adjuvant therapy is associated with various factors [47] including the histological subtype. The difference in the biological response to the various therapy between ASC cells and conventional SCC cells has been totally undetermined. Thus, our comparisons between ASC and conventional SCC are not accurate. However, we found that the ASC of the oral cavity may have poorer OS and DFS compared to both ASC cases of the head and neck region other than the oral cavity and conventional SCC cases.

To our knowledge, the present case is the only case of ASC with acantholytic features arising in the oral mucosa among reviewed cases. Although detailed pathological findings were unavailable for most of the cases in the literature reviewed, mucinous cells were observed in only 10 cases. Cases without mucinous cells were diagnosed based on glandular structures. Lymphatic and vascular invasion was observed in only three cases. Perineural invasion was observed in 11 patients. Age, TNM category, and surgical treatment were independently associated with OS, advanced T stage, distant metastases, and surgery for disease-specific survival [48]. However, the histological factors affecting prognosis, such as the percentage of glandular differentiation, are unknown.

In conclusion, we report that ASC consists of a squamous component with acantholytic features and an adenocarcinoma component with mucinous cell differentiation. Because ASC is a malignant neoplasm with a poor prognosis, a precise and accurate histological diagnosis is essential to estimate a patient’s outcome. Intracytoplasmic mucin can be a clue for diagnosing ASC, and special staining or immunohistochemical analysis for multiple markers to confirm glandular or mucinous differentiation is recommended. Although the mucinous component is limited to only a small area of the primary lesion, we recommend describing it in the pathological report and close clinical follow-up.

## Figures and Tables

**Table 1:** A comprehensive review of cases of adenosquamous cell carcinoma in the oral cavity.

Age	Sex	Site	Intraoral Findings	Histological Feature of Adenocarcinoma Component	Lymphatic/Vascular/Perineural Invasion	Treatment	LN Metastasis at the Initial Operation	Local Recurrence (Months)		Regional LN Recurrence (Months)		Distant Metastasis (Months)		Outcome (Months)		Reference
77	M	lower gingiva	indurative ulcer	glandular, mucus cell, acantholytic	Ly−, V+, Pn−	SO → SO, ND, RT	NE	+	13	+	13	−		AWOD	31	the present case
50	M	tongue	an ulceroproliferative lesion with induration	glandular	NA	NA	NA	NA		NA		NA		NA		[12]
70	M	floor of the mouth	a painful nodule with induration	glandular	NA	NAC → SO → SO, ND, CRT	NE	+	9	+	9	−	NA	DOD	14	[13]
48	M	tongue	a painless mass	glandular	NA	SO	NE	−		−		−		AWOD	9	[14]
51	F	tongue	a painful ulcer	glandular, mucous cell	Ly+, Pn+	NA	NA	NA		NA		NA		NA		[28]
65	M	tongue	well-defined indurated ulcer, with a leukoplakic lesion	glandular, mucous cell	Ly+, V+, Pn+	SO, ND, CRT	+	−		NA		+	6	AWOD	30	[15]
47	F	maxilla	nodular lesion	glandular, mucin	Pn+	SO, ND, RT → SO → SO	+	+	4 & 8	−		−	72	AWOD	72	[21]
97	F	maxilla	ulcer	glandular, mucous cell	NA	SO	NE	−		−		−		AWOD	12	[29]
59	F	lower gingiva	ulcer	glandular, pseudo-ductal	NA	SO → CRT	NE	+	12	NA		NA		DOD	23	[29]
55	M	upper gingiva	swelling	ductal	NA	NA	NA	NA		NA		NA		NA		[30]
62	M	tongue	NA	NA	NA	SO	NE	−		NA		NA		AWOD	17	[16]
59	F	tongue	NA	gland	NA	SO → ND	+	NA		NA		NA		DOD	11	[16]
63	M	tongue	NA	NA	NA	SO, ND?	−	−		NA		NA		AWOD	32	[16]
82	M	tongue	NA	NA	NA	SO, ND	+	NA		NA		NA		DOD	6	[16]
55	F	tongue	NA	gland	NA	SO, ND → CT	−	−		NA		NA		AWOD	17	[16]
61	M	tongue	NA	NA	NA	SO, ND	−	−		NA		NA		AWOD	19	[16]
83	M	tongue	NA	NA	NA	SO, ND	+	−		NA		NA		AWOD	22	[16]
71	F	tongue	reddish tumor with hemorrhage and erosion	glandular, mucous cell	Ly+, V−	SO	NE	−		−		−		AWOD	1	[17]
71	NA	floor of the mouth	NA	NA	NA	SO, RT	NA	+	NA	+	NA	+ (lung, bone)	NA	DOD	7	[31]
60	NA	floor of the mouth	NA	NA	NA	SO, RT	NA	+	NA	−	NA	+ (lung)	NA	DOD	9	[31]
68	NA	tongue	NA	NA	NA	SO, RT	NA	−		−		−		AWOD	10	[31]
60	NA	floor of the mouth	NA	NA	NA	SO, RCT	NA	−		−		−		AWOD	62	[31]
42	M	tongue	NA	NA	NA	NA	−	NA		NA		+ (mediastinum)	NA	DWD	34	[32]
65	F	tongue	NA	NA	NA	NA	−	NA		NA		−		NA	NA	[32]
48	M	tongue	NA	NA	NA	NA	+	−		NA		−		AWOD	3.5	[32]
87	F	tongue	NA	NA	NA	NA	+	NA		NA		−		AWD	9	[32]
62	M	soft palate/tonsil	NA	NA	NA	NA	+	NA		NA		+ (neck skin)	NA	DWD	36	[32]
70	M	upper and lower gingiva	swelling	glandular	NA	RT	NE (clinically +)	NA		NA		+	at the initial	NA		[33]
42	M	mouth	NA	ductal, mucous cell	NA	NA	NA	−		NA		NA		AWD	20	[18]
54	M	mouth	NA	ductal	NA	NA	NA	NA		NA		NA		AWD	36	[18]
68	M	tongue	NA	ductal, mucous cell	NA	NA	NA	−		NA		NA		AWOD	56	[18]
43	M	mouth	NA	ductal, mucous cell	NA	NA	NA	NA		NA		NA		DOD	12	[18]
72	M	floor of the mouth	ulcerative elevation	ductal	NA	SO, ND	+	−		−		−		DWOD	60	[34]
22	F	tongue	ulcer	ductal	Pn+	SO, ND → SO	−	+	9	NA		NA		DWD	12	[19]
38	M	floor of the mouth	NA	NA	Pn+	SO, ND, RT	+	NA		NA		−		NA		[23]
80	M	floor of the mouth	NA	NA	Pn+	SO, RT → NA	+	+	12	NA		−		AWOD	47	[23]
59	F	floor of the mouth	ulcer	NA	Pn+	SO, ND, RT	+	+	9	+	NA	−		AWD	31	[23]
74	M	floor of the mouth	ulcer	NA	Pn+	SO, RT	NE	+	12	NA		−		DWD	24	[23]
34	F	palate	plate mass	NA	Pn−	SO, RT → NA	NE	−		NA		−		AWOD	78	[23]
67	M	lower gingiva	ulcer with raised edge	ductal	Pn+	RT	NE	−		−		−		NA	NA	[35]
46	M	floor of the mouth	indurated, ulcerated mass	glandular, mucous cell	NA	SO, partial ND	−	−		−		−		AWOD	6	[35]
65	F	tongue	indurated ulcer	ductal structure	V+, Pn+	SO	NE	−		−		−		AWOD	4	[35]
78	F	tongue	diffuse reddish swelling	glandular	NA	CRT	NE (clinically +)	NA		NA		NE		DOD	4	[20]
42	M	tongue	painful ulcer, erythroplakia	ductal	Pn+	SO, ND	−	NA		NA		NA		NA		[36]
61	F	upper gingiva	swelling	glandular, mucous cell	NA	SO, RT	+ (FNA)	NA		−		NA		DOD	6.5	[37]
54	M	tongue	NA	NA	NA	SO, CT	NA (Non-regional +?)	NA		NA		+	NA	DOD	9	[2]
53	M	tongue	NA	NA	NA	SO, ND	NA (Non-regional +?)	NA		NA		+	NA	DOD	180	[2]
60	M	tongue	NA	NA	NA	SO, ND	NA (Non-regional +?)	NA		NA		+	NA	DOD	12	[2]
47	M	floor of the mouth	NA	NA	NA	SO → SO, ND	+	+	36	+	36	−		DOD	36	[2]
57	M	floor of the mouth	NA	NA	NA	SO → SO, ND	+	+	3	+	3	−		AWD	8	[2]

Abbreviations: M, male; F, female; Ly, lymphatic invasion; V, vascular invasion; Pn, perineural invasion; SO, surgical operation; ND, neck dissection; CRT, chemoradiotherapy; RT, radiotherapy; CT, chemotherapy; FNA, fine-needle aspiration; LN, lymph node; AWD, alive with disease; AWOD, alive without disease; DOD, dead of disease; DWD, dead with disease; DWOD, dead without disease; NE, not evaluated; NA, not available; +, positive; −, negative.

**Table 2:** Summary of the literature review.

Age		22–97 (Median 60)	
Sex	male	31	62.0%
	female	15	30.0%
	unknown	4	8.0%
Site	tongue	25	49.0%
	floor of the mouth	10	19.6%
	upper gingiva	5	9.8%
	lower gingiva	4	7.8%
	palate	2	3.9%
	not specified	5	9.8%
Locoregional recurrence		27/43	62.8%
Local recurrence		12/23	52.2%
Lymph node metastasis		20/43	46.5%
	at initial operation	17/43	39.5%
	regional lymph node recurrence	6/43	14.0%
Distant metastasis		9/30	30.0%

## Data Availability

Data sharing is not applicable to this article.
